# Ebola impact on African health systems entails a quest for more international and local resilience: the case of African Portuguese speaking countries

**DOI:** 10.11694/pamj.supp.2015.22.1.6653

**Published:** 2015-10-11

**Authors:** Luís Velez Lapão, Andreia Silva, Natália Pereira, Paula Vasconcelos, Cláudia Conceição

**Affiliations:** 1 Global Health and Tropical Medicine, Instituto de Higiene e Medicina Tropical, Universidade Nova de Lisboa, Portugal; 2 Direcção Geral da Saúde, Ministério da Saúde, Portugal

**Keywords:** Ebola, impact on health systems, resilience, crisis coordination

## Abstract

**Introduction:**

Ebola epidemics have shown to have significant impacts on many aspects of healthcare systems. African countries have been facing many difficulties while addressing Ebola epidemics, moreover due to both lack of resources and fragmented involvement of national and international entities. The participation of multiple organizations has created serious problems of coordination of aid and the operation of that aid on the ground. This paper aims at addressing the impact of Ebola epidemics on African health systems, with a special focus on the definition of impact mitigation guidelines and the role of resilience. The example of Portuguese speaking countries is presented.

**Methods:**

A combination of literature review and case study methods are used. A literature review on Ebola outbreak impact on health systems will provide information to define a set of guidelines for healthcare services response to Ebola. The role of cooperation in providing additional resilience is described. Finally a case study focusing on the Portuguese collaboration and intervention in African Portuguese Speaking Countries (PALOP) is presented, as an example how the international community can provide additional resilience.

**Results:**

The existing knowledge is very helpful to guide both the preparation and the coordination of Ebola preparedness interventions. Additional resilience can be provided by international cooperation.

**Conclusion:**

In addition to international concrete support in times of crisis, to have a regional strategy of creating (multi-national) teams to rapidly implement an intervention while establishing better regional capacity to have sufficient resources to support the “resilience” required of the health system.

## Introduction

The Ebola virus arrived in the end of 2014 to western countries (the United States [1] and Spain [2]) creating a buzz in the media, but it has been mostly confined to western and central Africa. The recent outbreak emerged in this zone in March 2014 [3]. Most of these countries health systems have weaknesses that hinder a quick and efficient intervention to respond to the high mortality rate, ranging from 25 to 90% [4, 5]. The World Health Organization (WHO) has been the main coordinator and mobilizer of interventional action, together with the institutions of the affected countries and with the support of some NGOs, which the Doctors-without-Borders (MSF) is among the most actives. Only after the threat has become clear, the United States and England set up field hospitals in the most affected areas. However, this fragmented involvement of national and international entities of multiple authorities has created serious problems of coordination of aid and the operation of that aid on the ground. Portuguese Speaking countries in Africa were not directly affected by Ebola; but they needed to address the eventuality of the arrival of the disease.

## Methods

This papers aims at identifying the most important Ebola outbreak impact factors on the African health systems. The paper addresses the main healthcare services topics and provides guidelines to mitigate health services impact. Look at what happened in Africa, and what can we learn from it. A combination of literature review and case study methods are used. A literature review on Ebola outbreak impact on health systems will provide information to define a set of guidelines for healthcare services response and organization in such cases. The description of set of international collaboration cases will address the role of cooperation and resilience support. Finally a case study focusing on the Portuguese collaboration and intervention in African Portuguese Speaking Countries (PALOP) is presented, as an example how international community can provide additional resilience. The case study includes the collaboration of both Instituto de Higiene e Medicina Tropical (IHMT) and DireçãoGeral de Saúde (DGS, National Health Directorate) with Cape Verde and Guinea-Bissau.

## Results

### Literature review

Epidemic outbreaks frequently threatenboth social organization and healthcare services. There is limited literature on causes of impact on health services. What is known today about Ebola is mainly due to past epidemics [6, 7], recent brief articles and some local research and the media. Epidemics are always situations that have the potential to cause instability, and sometimes even some panic in the populations. Despite the fact that epidemic soften leads to the creation of strategic crisis centers, usually installed in facilities outside health facilities, these are not without impact, especially because the health facilities are ill-prepared and vulnerable, with few resources and equipment. The evidence shows that the Ebola epidemic produces increased pressure on health professionals, with impact on the delivery of health services and may lead to disruption of the same and eventually the closure of hospitals and health centers[5, 8]. Ebola creates also impact on the population's behaviour [9]. It generates an increased on the demand for health services (with patients often left at hospital's door to avoid entering into the building [10], but there were reported cases of patients fleeing services (e.g. in Guinea). This happens due to the perception of increased risks and because the healthcare services cannot give agood clinical or nourishment answer. There have also been identified community participation and commitment problems that affect the success of isolation measures [4]. It has been reported that children and pregnant women, for fear or because there are no services available, end-up staying at home, often without the possibility of accessing even the simple treatments [11]. Others sought traditional medicines [12] and often seek services only have a very serious condition [10]. There were situations of conflict and with some violence, leading to the need for intervention by the authorities. Often this violence is directed at health professionals, or even against members of the population, that after infected survived the disease. One of the most significant pressures is affecting health professionals’ activities. The information available has shown a significant impact of the Ebola epidemic in work organization, affecting both the health and daily lives of health professionals in the affected countries. For example, Liberia has only about 50 doctors and 1000 nurses to 4.2 million people, and in Sierra Leone just over 135 doctors and 1017 nurses for a population of 6 Million inhabitants [13]. It is very difficult for the healthcare systems to respond without weakening the response of other services, often left without capacity. UNICEF Liberia has warned that the effort to deal with Ebola created disruptions in paediatrics [14]. In various locations, health professionals do not attend the services terrified to be infected with the Ebola virus [10]. A significant percentage of those infected are health professionals [9, 15], which not only limits the answer directly, but also disrupts the recruitment of more professionals. Often these infections result from not using biosafety equipment, or from its careless use. There are problems with follow-up procedures or lack of training in the use of PPE [2]. The lack of rigor in the technical approach has caused a growing distrust and alarmism in populations. Healthcare services access are also deeply affected. Significant impact results from the pressure to diagnose epidemic diseases, which often leads to under-diagnosis of the most prevalent and endemic diseases, resulting in an increase of preventable mortality, such as malaria or cholera. This trend is accentuated by the collapse of immunization services. There are suspicions that, in Liberia, the number of cases identified as Ebola includes cases of cholera, malaria and typhoid fever [11]. The efforts of the Ebola affected the access of other patients that with the fear of contact health services they stay at home, which can lead to increased mortality in the health system associated with these cases. As mentioned, the collapse of prevention and health promotion services also limit children′s access to immunization services and pregnant women to maternity hospitals where they can have their children safely. An Ebola epidemic creates additional transport and logistics needs that will affect the few commonly available resources and the distribution of donations received from abroad. Transportation of patients to treatment centers, drugs and materials, food and water will require coordination to ensure adequate and timely supply. In crisis situations, the military can make an important contribution combining logistical support safely. Reuters [16] reported difficulty in safely transport vaccines in Africa, mainly due to lack of logistical capacity to keep vaccines cold. To support a strategy to keep monitoring the chronically ill in their homes, reducing the pressure on hospitals and avoiding possible exposure to the virus, it is necessary to plan additional reinforcement means for transporting these health professionals to patients’ homes. There are still the logistical problems for accessing food and other essentials products, resulting in significant impacts on local markets that are affected by reduced supply and consequent price increase. This further exacerbates the effort required by the health services to be supplied conveniently. Epidemic's control intervention and coordination Impacts are significant. Given the limited capacity and the poor preparation of local health services, the response of international agencies and humanitarian organizations, in the context of a weakness of local or regional leaders, end-up creating multiple coordination centers and controlling the activities. This leads to disparate approaches that increases the inefficiency of the responses and creates an “anxiety” epidemic [17].

### Guidelines to reduce the impact the healthcare system

Health systems resilience is important to respond to an epidemic of Ebola [5]. The notion of resilience includes the ability to adapt and face resistance from a challenge, which for healthcare organizations point of view implies the existence of some redundancy to respond to crises. In Africa this resilience is often only possible by sustained international cooperation.

### What to do to face an Ebola epidemics and before existing confirmed cases

A key point is to ensure the preparation of coordination teams [5]. The response strategy should privilege the training of health professionals at all services levels; to seriously prepare them through drills and cooperation exercises between entities to allow filing potential gaps. One should take the opportunity to validate the existence of defined and implemented processes in accordance with the guidelines. These validation processes need to be taken with watchfulness. Another critical aspect is to ensure the professionalism of coordination and all procedures; the risks are too serious to be otherwise. The infection control committee, in collaboration with the management of health facilities at all levels, and with the coordination of central entities, should consider cooperation and flexibility mechanisms. To this end, the following questions should be strictly considered as a guideline [18]: is there a “National Contingency Plan for Ebola”? If so, where can it be found?

Does this plan addresses the issues related with communication of citizens with health services and health services with the media?

Is the Ebola action role of each health service defined within the National Health System (public and private); and how to articulate them together?

Is the national public health service network already prepared to perform epidemiological surveillance?

Is identified who will proceed to the epidemiological investigation of reported cases and their contacts? Is there a plan to protect these professionals and to give them proper working conditions?

What is the role of laboratories and how are they prepared and integrated within the contingency plan? Is the national reference laboratory prepared to deal with this issue, particularly with biological safety aspects it entails? Is there a plan to protect these professionals?

Is available in the hospital, the treatment currently considered reference to treat the disease? If so, how many people can be treated? What treatment options are currently available in the country?

It has been made available training for the professionals who will deal directly with patients? If so, are enough professionals available to face the risks of high-transmissibility suspected virus and its high lethality?

Have the authorities provided the mechanisms for the regulation and control of the use of protocols and security procedures at reference hospitals and have other health facilities been identified? Have the relationship between the Ministry of Health and Emigration Services and Borders been defined, and will updates be provided aboutpeople entering from high-risk countries? Have these professionals provided with the adequate training to deal with such cases? Has been provided accurate information about people entering the country? What mechanisms exist to detect potential infected at entry points?

### Healthcare services organization

The infection control committees (ICC) often are responsible for the coordination of health facilities response efforts, in particular for the development, implementation and monitoring action protocols. These efforts are to create supportive conditions and orientation of potentially infected cases, the maintenance of hygiene and cleaning, and for ensuring that essential services are maintained operational [8, 19]. The following health services as essential and they must be provided with all the conditions to be kept operational [8]: maternity; emergency; pediatrics; internal medicine; essential surgery; and trauma; coordination, at institutional level, should ensure proper organization of healthcare professionals’ work in order to avoid overwork, which often lead to error that can have serious consequences. An important aspect in view of streamlining the access to the hospital for chronically ill (including cases of TB and HIV), it is advisable that can be done home treatment of patients. Such an approach helps if you have the support of Primary Healthcare. The ICC must adapt procedures to avoid the spread of the Ebola virus within health services and to minimize the disruption of the normal functioning of health facilities.

### Services coordination and integration

Coordination and integration of services are often delivered by the National Health Directorates, which should ensure a clear flow and a systematic recording of all necessary information, as well as their timely sharing. In West African countries, hospitals end-up having an important role in the process of sorting and sometimes diagnostic and should be prepared to make it so [8]. The first measure should be the creation of a “green line” to easy the communication with the population. The national coordination also plays an important role in the management of human resources to agile the response to the epidemic and to avoid disruptions in essential services, especially considering the high number of teams that may exist on the ground if engaged on the primary health care services, following the chronically ill. To promote better coordination between health services is advised that [8]: hospitals and health centers boards should be involved in the overall Ebola planning efforts; The ICC should also include managers of these units to facilitate the mobilization of resources. They should meet regularly to monitor planning and implementation activities; Develop a set of interventions to improve the infection control (supervision, training, early screening and case detection, disinfection, transfer of suspected cases and hygiene improvement measures).

### Safety and logistics

The MSF [8] presents a comprehensive list of activities to be undertaken to assess the responsiveness of logistics potential affected area: evaluate existing logistical resources within the community and develop a list of missing items, including road and water transport, fuel availability, etc; check the stock of available equipment (personal protective equipment (PPE), disinfectants, drugs, etc.) and sterilization equipment capacity within the community or nearby; assess communications and record locations in GPS (geo-referenced roads, radio, telephone, airstrips, critical material, etc); gather maps of the affected area or from locally available sources or from relevant government agencies; assess accommodation available in the area, water, electricity and food for the response teams; assess availability of local human resources (health professionals, community health workers, volunteers of the Red Cross, NGOs, and technical advisers will operate in the affected area, etc); visit and evaluate health facilities (the number of beds, crematoria, cold chain capacity, access to clean water, electricity, space for the creation of an isolation area, storage space) and identify possible modifications that need to be performed. Also evaluate the behaviour of the demand for health care, such as traditional healers; identify any socio-cultural contexts that can complicate the operation; assess the security situation in the area. The security situation should be reassessed regularly. Furthermore, recommended the establishment of a logistics team and security in order to provide logistical support in operations and ensure the safety of teams in the field [20].

### The role of information management

A crucial aspect, which has been shown as a weakness in various circumstances, is the management of information. It is necessary to establish a clear flow of information to avoid the risk of wrong decisions, incomplete records or double counting, and to ensure that information is shared appropriately and in a timely manner to allow a quick and correct response [9]. Information management needs to be taken seriously in order to maintain privacy and protect patients, and their families, avoid social stigmatization potential as well as taking into account the ethical aspects. Another important point is to organize the ability to record information freeing health professionals to do so, since they will be busy and with major limitations to register it conveniently [8].

### Communication strategy

The Ministries of Health, and crisis coordination teams should seek to communicate “with one voice”, clearly and in an assertive manner, for example, through a respected and credible spokesperson [9]. The broad scope of the media makes them key partners, which should be treated professionally and on a basis of trust to avoid transmitting information that could destabilize the response on the ground. The mobile telecommunications networks can be enhanced as access to privileged information mechanisms, either as communication between people and coordination team, or a communication between the teams [20]. The Economist [21] suggested the possibility to use mobile communications to better understand the social and health service demand dynamics associated with Ebola epidemic. However, there must be a coordinator of communications, establishing mechanisms to rapidly exchange information between the various teams and the media, to avoid rumours and misinformation, and to promote the sharing of information that is easily understood. An assertive communication mechanism also helps to demonstrate to the public that local authorities are responding to concerns of local communities. A professional communication team should develop a communication plan, together with the Ministry of Health and other Ministries, in order to convey coherent and comprehensive messages about the action on the ground;

### Cooperation with african Portuguese speaking countries

For the first time in history, the United Nations adopted a resolution establishing an emergency mission in public health (United Nations Mission for Ebola Emergency Response - UNMEER) to combat the epidemic of Ebola, whose outbreak takes place in West Africa since March 2014. This resolution joined governments and international partners on a global response. The WHO recommendations stressed that all countries with land borders with the affected countries, should: establish access to qualified laboratory diagnosis for the disease Ebola virus; ensure that health professionals are adequately trained on prevention and infection control; establish rapid response teams equipped to study and manage cases per Ebola virus. Other strategies include UNMEER support the countries collaborative efforts in the mechanisms of prevention and response to disease Ebola virus, with special focus on human resources training. Missions in the Republic of Guinea-Bissau: Considering the prevention of cross-border spread of the virus, the Portuguese authorities were contacted by UNMEER to listen on the availability of Portugal participating in the initiatives in Guinea-Bissau, which has land border with Guinea. Given the mutual interest, within the bilateral cooperation context, Guinea-Bissau and Portugal signed the Action Plan (November 2014 to June 2015). This plan aims at supporting Guinea-Bissau in the health sector, is coordinated by the Camões - Cooperation Institute and Language. Based on the strategies defined by UNMEER, the initiative is conducted under the Inter-ministerial Commission for the Coordination of Response to Ebola, and includes, General Health Directorate (DGS), the National Institute of Medical Emergency (INEM) and the National Institute of Health Dr. Ricardo Jorge (INSA). This specific initiative in Guinea-Bissau includes the multilateral level in partnership with WHO. The first stage involved a visit to assess local needs, followed later by a second visit of Portuguese entities together with WHO to assess the health and structural conditions existing in that country as part of prevention and response to Ebola virus disease. These visits found that despite the efforts and improvement of Guinea-Bissau conditions in recent years remain many local difficulties in the prevention and response to Ebola virus, as the result of various constraints: lack of adequate financial resources and logistics; lack of human resources trained to identify and to treat such patients; no existence of a laboratory to detect the Ebola virus. The latter aspect was identified as crucial, in view of the lack of laboratory facilities with the necessary biosafety levels, moreover since it will not be feasible handling and shipping samples to WHO reference laboratory in Dakar. In this bilateral support package for Guinea-Bissau, Portugal provided support to the City Council Bissau on awareness activities of the population and combating the spread of the Ebola virus, by adopting preventive measures. This has included sending disinfectants, PPE, medicines and food goods. It also included the training of a Guinean technician in the transport of Infectious Substances. In this regard, a mobile laboratory will be provided and a multidisciplinary Portuguese team will run its operation. It is expected to begin operating during the month of March 2015, and maintained throughout 2015. It is intended to create conditions contributing to the early detection, laboratory diagnostics, rapid response and implementation of a surveillance system to ensure that local treatment of possible cases of disease Ebola virus and prevent the evacuation of infected patients. This intervention had an additional positive impact by allowing the resumption of air links between Portugal and Guinea-Bissau. From the resumption of air links with Guinea-Bissau on November 14th, 2014, it was implemented a screening to passengers at the exit of the country, consisting of the evaluation of body temperature, meeting the screening criteria in exit points. This screening has been carried out by INEM technicians for the early detection of possible signs and symptoms suspicious of Ebola virus. The procedure takes place in conjunction with the Guinean authorities and the Foreigners and Borders Service of Portugal, happening regularly on all flights made. It is expected to maintain this procedure while during the Ebola outbreak in the western coast of Africa. This particular activity has elapsed uneventful and to date, have not been identified patients leaving this country to Portugal. Mission in Cape Verde: Upon the request of the Central hospital of Cape Verde, a team from IHMT (included an infectiologist and a public health expert) visited the hospital and during two days revised, with the hospital responsible team and with the national public health authorities, the contingency plans concerning the possibility of an Ebola outbreak. The bulk of the work was the discussion of a multitude of operational details. The circuits of patients through the hospital, from the different points of entry of potential infected patients to the isolation ward, were also revised, as were the isolation premises and the circuits of the different critical materials. The use of PPE was a matter of great concern. Adding to this, in the fall of 2014, DGS sent personal protective equipment biosafety level 4. Portugal has also been working with other African Portuguese speaking countries. INSA experts working with São Tomé e Principe health authorities exchanged preliminary information of technical, equipment and human resources requirements, in order to evaluate the capability for safely sending samples to INSA, as the reference laboratory for Ebola analysis. The Ministry of Health of Portugal sent biosafety level 4 PPE to Mozambique. A course in virology was held in Maputo in October 2014.

## Discussion

Several complementary factors contribute to stop the threat of transmission of Ebola: leadership, organization of services, coordination and communication, logistical support and a set of quick actions. Good communication of coordinating entities to health facilities is key. In hospitals infection control commissions plays an important role in the coordination of procedures. Coordination teams should give special attention to highly vulnerable health professionals, usually ending-up to be a significant proportion of infected [5]. The coordination should look for the right conditions to maintain essential services and provide equipment usage training in the interaction with potential patients. To avoid disruption of health services, it should be identified the appropriate channels for patients with symptoms associated with Ebola (under the guidance of local authorities). Nigeria has been considered a good example of action and coordination. WHO confirmed on 20 October 2014 that Nigeria had come to control the epidemic of Ebola. Is attributed to the success of Nigeria by “avoiding a worst-case scenario” the “quick and forceful” answer [22]. These authors point to three key elements in its approach as “epidemiological detective” and rigorous enforcement of public health practice: rapid and complete identification of all potential contacts (about 900); continuous monitoring of all such contacts; the rapid isolation of all potentially infectious contacts. The rapid launch of measures often requires the participation of international collaboration. In the case of African Portuguese speaking countries, these measures were taken in preparation before cases were identified, contributing to a higher resilience to address the epidemic, if it has happened.

## Conclusion

The response to Ebola, in African countries with limited resources, it is often very demanding: the shortage of qualified staff, lack of equipment and adequate facilities and equipment (i.e., thermometers, PPE, disinfection detergents, laboratory equipment, equipped rooms, etc.) hinder a good and effective response. It will be crucial, in addition to international concrete support in times of crisis, to have a regional strategy of creating (multi-national) teams to rapidly implement an intervention while establishing better regional capacity to have sufficient resources to support the “resilience” required of the health system. Each national team of rapid response should include an epidemiologist, a doctor, a specialist in laboratory, a logistics and communications coordinator, and other necessary experts in infection control, anthropology, social mobilization, geographer, veterinarian, etc. [21].
